# Combining Deroofing with LAight^®^ Therapy for Long-Term Disease Control of Hurley Stage II Hidradenitis Suppurativa: Case Report

**DOI:** 10.3390/clinpract11010005

**Published:** 2021-01-27

**Authors:** Michael Schultheis, Margot Scheuern, Ivan Radkov

**Affiliations:** 1 Department of Dermatology, University Medical Center of the Johannes Gutenberg University, 55131 Mainz, Germany; 2 WZ®-WundZentren GmbH, 40217 Dusseldorf, Germany; Margot.Scheuern@wundzentrum-duesseldorf.com; 3 MVZ Mgefäss und wundVersorgungsZentrum GmbH, 47119 Duisburg, Germany; ivanradkov@t-online.de

**Keywords:** case report, deroofing, LAight therapy, hidradenitis suppurativa, acne inversa

## Abstract

There is an increasing consensus that the treatment of Hidradenitis suppurativa (HS) significantly varies with the degree of inflammation and that treatment according to guidelines is not always successful. Here, we report the case of a 31-year-old male with widespread, highly inflammatory Hurley Stage II HS in multiple locations who failed to respond to any kind of established medical treatment, including biologics. As an alternative approach, Ustekinumab was maintained, and additionally the patient was treated with LAight therapy, a combination of intense pulsed light and radiofrequency. After 10 sessions, deroofing of multiple lesions was performed in a two-step process. After a few weeks of healing time accompanied by specialized wound experts, the patient continued with LAight therapy to control and prevent recurrence. This case shows that the combination of LAight therapy and deroofing is a promising treatment plan for the long-term symptom control of mild and moderate HS.

## 1. Introduction

Hidradenitis suppurativa (HS), also called Acne inversa (Ai), is an inflammatory skin disease of the inverse regions with an extreme burden for patients. In moderate-to-severe disease, it leads to the formation of scarring and irreversible tissue destruction in the affected areas. Lesions are associated with pain and drainage of malodorous secretions, resulting in a significant impact on quality of life [1,2].

Mandatory indications for surgery represented by irreversible tissue damage have recently been defined and published [3]. However, the authors suggest that in case of remaining inflammatory lesions, a combination with medical treatment options should be pursued [3].

Medical treatments include topical and systemic antibiotics, hormonal agents, and biologic medications [4]. These therapies can be successful in controlling the disease, yet discontinuation is often associated with relapse of symptoms and the risk of antibiotic resistances [5,6,7]. Wide surgical excision can induce long-term symptom control but may not be appropriate for all patients considering the long, cumbersome healing process [8].

The deroofing technique is an effective and fast surgical technique with maximal preservation of the surrounding healthy tissue, especially suited for recurrent HS lesions at fixed locations in Hurley I or II areas. However, since HS is a chronic disease with frequent episodical flares, studies show a recurrence rate of 17% of the deroofed lesion itself, as well as the risk of formation of new lesions in the treated area [9].

Consequently, noninvasive treatment options which can induce long-term symptom control are needed. The LAight^®^ therapy (LENICURA, Wiesbaden, Germany) is a device-based therapy combining intense pulsed light (IPL) and radiofrequency (RF), which is applied locally to the affected skin areas (for technical characteristics see Table 1). According to the studies, this leads to a reduction in inflammatory lesions and a significant increase in quality of life as measured by the Dermatology Life Quality Index (DLQI) [10,11]. The device obtained CE-certification for all stages of HS in 2017.

In the following, we report the case of a 31-year-old male with widespread, highly inflammatory Hurley Stage II HS in multiple locations who failed to respond to any kind of established medical treatment, including biologics, and whose disease could be effectively controlled under a combined treatment plan of deroofing and LAight.

## 2. Case Presentation

**Figure 1:**
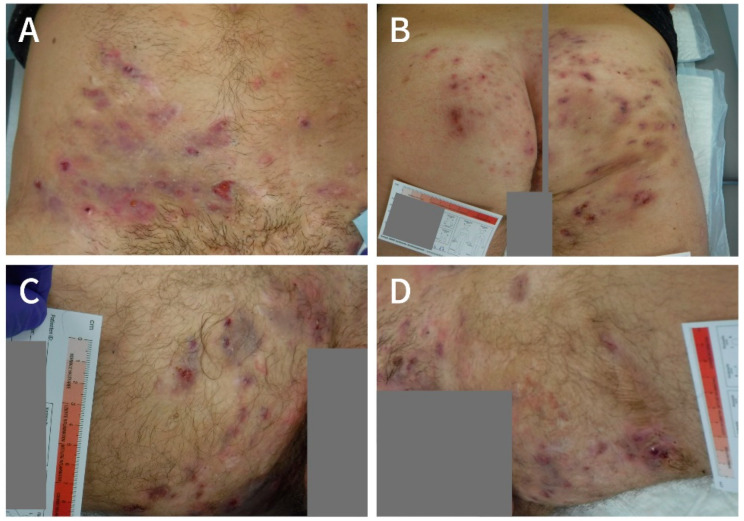
Manifestations of Hidradenitis suppurative (HS) in the mons pubis area and lower abdomen (**A**), buttocks (**B**), right thigh (**C**) and left thigh (**D**) at the beginning of LAight therapy (July 2017).

**Figure 2:**
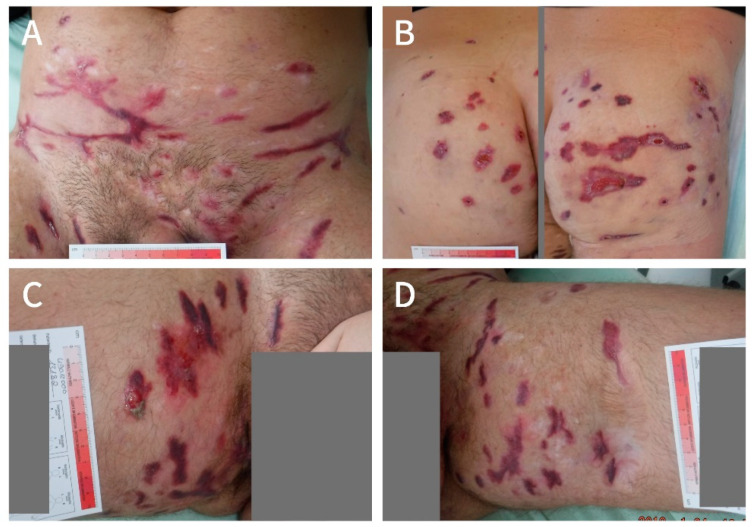
Manifestations of HS the mons pubis area and lower abdomen (**A**), buttocks (**B**), right thigh (**C**) and left thigh (**D**) after deroofing (January 2018).

**Figure 3:**
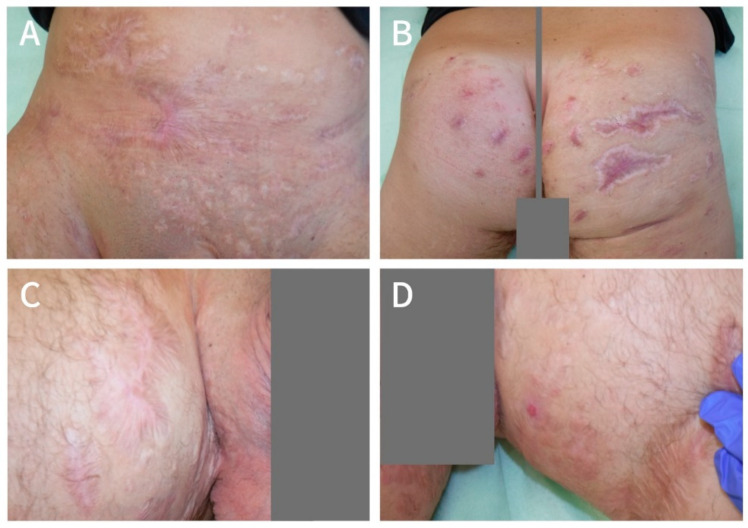
Manifestation of HS the mons pubis area and lower abdomen (**A**), buttocks (**B**), right thigh (**C**) and left thigh (**D**) after additional 25 months of regular LAight therapy (April 2020).

In July 2017, a 31-year-old male patient presented to the WZ-WundZentrum in Dusseldorf, Germany, with a widespread Hurley stage II HS in both thighs and groins as well as in the mons pubis area, lower abdomen and buttocks (Figure 1A, Figure 2A and Figure 3A). He was a light smoker (three cigarettes per day), had a BMI of 27 kg/cm^2^, and reported no relevant comorbidities.

According to the patient, the first symptoms appeared in January 2009 and it took more than 6 years until he received the proper diagnosis in November 2015, in a dermatologic clinic specialized in HS.

In the very beginning of the disease, he had a wide excision with consecutive vacuum pump therapy on the left thigh which left him considerably traumatized, since postoperative care was associated with severe bleeding and pain.

Further treatment history included the guideline-recommended routine of antibiotics with Clindamycin and Rifampicin for 10 weeks and Triclosan 2%, which failed to control symptoms. In the years 2014–2015, multiple incisions were performed.

After having received the proper diagnosis, the patient was started on Adalimumab (40 mg weekly) in February 2016. Since Adalimumab did not show much effect, Cefuroxim was additionally given for 4 weeks in July 2016 and later, in November 2016, Colecalciferol and Prednisolon were added. Interestingly, the patient responded well to steroids but after termination, symptoms recurred quickly.

After one year of weekly injections, Adalimumab was terminated, and the patient was started on Ustekinumab in February 2017, which was combined with 6 weeks of Ertapenem in March 2017. Still, the effect was limited, and so Prednisolon was given again in February 2017, which improved the disease, but after termination, symptoms almost instantly recurred.

When presenting at the WZ-WundZentrum in Dusseldorf in July 2017, the patient was severely impaired by his disease (Figure 1) with a reported DLQI of 22 and a pain score of 5 on the numeric rating scale (NRS). The restrictions affected his private and professional life, since he was not able to work in his job and although he was in an intact relationship, the fact that he regularly could not take part in social activities was burdensome to him. The German social system granted an official disability status in March 2017 (“Grad der Behinderung” of 50). Moreover, the consistent pain required permanent usage of potent analgesics like Metamizol.

Finally, the patient feared another surgical intervention with wide excision of tissue, since his past experiences had been traumatizing.

In July 2017, the patient was started on biweekly LAight therapy in addition to his ongoing Ustekinumab injections.

In November 2017, after 10 LAight treatments, the lesions had become more superficial, but the patient’s condition still did not allow him to work or participate normally in social life (DLQI of 23 points) and the whole situation imposed increasing mental strain.

Because the lesions did not show intensive scarring and were mainly distinct, a two-step deroofing procedure (first step: groins, thighs, mons pubis, and lower abdomen; second step: buttocks) was proposed by the surgeon in the dermatologic clinic specializing in HS. Since the patient was well educated about the difference between deroofing and wide excision and trusted the surgeon as well as the wound-care specialists who had been taking care of him during the preceding months, he consented to the procedure.

At the end of November 2017, multiple lesions in the groin, thighs, mons pubis area, and lower abdomen were deroofed, and in February 2018, deroofing was performed on the buttocks without complications (Figure 2). In between the two procedures, the patient continued LAight therapy on the buttocks prior to operation (three sessions).

To prevent infection after surgery, the wounds were cleaned three times a week with an antibacterial wound irrigation solution. With the method of the wet–dry phase and additional cooling, an itch-reducing effect could be achieved. Deeper, more secreting wounds were padded with a gel-forming fibre dressing. In combination with a polyurethane foam dressing, which has a high absorption and retention capacity, it was possible to dispense from daily dressing changes as well as to achieve a high degree of comfort. Due to the silicone coating of the dressing, the dressing changes were largely painless for the patient. The bandage was fixed with a waterproof fixing film which made it possible for the patient to shower. For the scarred and itchy areas, various skin care products with moisturizing or itch-reducing properties were used to strengthen the skin barrier and improve quality of life.

At the end of March 2018, the wounds had healed to a great extent and, at that point in time, the patient already felt a significant improvement in life quality with a DLQI of 14 points and pain NRS of 0 points. He restarted LAight therapy on a monthly interval to manage recurrence (as in Figure 2C) and to improve scarring. By that time, he had also quit smoking.

Since March 2018, the patient has continued his Ustekinumab therapy and still follows a constant LAight treatment routine. After seven monthly sessions, the treatment rhythm was extended to every 8 weeks. During that time, he had several recurrences in the operated areas, as well as one abscess in the right axilla, which all vanished under LAight therapy. With current treatment modalities, the level of active lesions is controlled at a very low level. Since he restarted his job in summer 2018, he did not miss a single day at work. In April 2020 (two years after surgery), the patient reported a DLQI of 3 points and a pain score of 1, implying that his disease only has a small effect on his life (Figure 3).

## 3. Discussion

The presented case shows that HS is a debilitating skin disease for which treatment can be challenging. The very painful course of the illness, the restricted mobility, the development of odour and the associated mental stress significantly reduced the quality of life of the patient.

The widespread, highly inflammatory lesions did not respond to any kind of established medical treatment. Steroids had a temporary positive effect on disease symptoms; however, they could not be applied as a continuous therapy.

We show that deroofing combined with LAight therapy can be a very effective long-term treatment plan for Hurley stage I and II patients. The two-step deroofing procedure was performed without complications and recurrence, and the formation of new abscesses in the axilla is well controlled with LAight.

In contrast to a wide excision, deroofing is associated with much less downtime and keeps the surrounding healthy tissue intact [9]. LAight can be carried out without anaesthesia and can easily be integrated into the daily life of patients. According to the manufacturer (LENICURA), in an acute phase of inflammation, treatment should take place every other week. After the acute inflammatory phase, to prevent recurrence, the interval between LAight sessions can be extended to 4–8 weeks depending on the severity and personal relapse of symptoms. As it is not a systemic treatment, no regular lab controls are needed, and, to date, mild local, temporary skin irritations are the only observed side effects [11]. According to studies, IPL and RF have physical properties that can be used positively for the treatment of HS, and Seok et al. recently pointed to a synergistic effect of the two components in a rabbit ear model [12]. It has been shown that IPL blue light has anti-inflammatory effects and can cause superficial desquamation of the epidermis, reducing the blockage of the hair follicles, which is a major cause of the development of HS [13]. This effect might be enhanced by the application of RF, which causes the liquefaction of trapped lipids, and thus also releases the blockage. Moreover, RF is known to increase the blood flow, and to stimulate collagen production, which improves scar texture [14]. We also observed this in our patient and for the last two years, he has been highly satisfied with the treatment outcome and regained full work productivity.

## Figures and Tables

**Table 1:** Technical characteristics of LAight therapy.

Treatment Passes	RF Intensities in J/cm^2^	Impulse Characteristics RF	IPL Wavelength Interval in nm	IPL Intensities in J/cm^2^	Impulse Characteristics IPL
1st treatment pass	12.2	1 impulse with 1 s duration and frequency of 1 MHz	420–1200	6.0	4 sub-impulses with 8 ms duration and 8 ms pause
2nd treatment pass	12.2	1 impulse with 1 s duration and frequency of 1 MHz	510–1200	5.6	4 sub-impulses with 8 ms duration and 8 ms pause
3rd treatment pass	12.2	1 impulse with 1 s duration and frequency of1 MHz	690–1200	4.4	4 sub-impulses with 8 ms duration and 8 ms pause

## Data Availability

All relevant data for the case report are presented in the text.
